# Cross-elbow stent placement for refractory arteriovenous fistula stenosis and chronic total occlusion in hemodialysis patients: a single-center retrospective cohort of 42 cases

**DOI:** 10.1080/0886022X.2026.2738994

**Published:** 2026-10-27

**Authors:** Yibiao Huang, Linnan Bai, Junnan Wu, Xuan Zheng, Mingxi Lu

**Affiliations:** Department of Nephrology, Sir Run Run Shaw Hospital, School of Medicine, Zhejiang University, Hangzhou, Zhejiang, China

**Keywords:** Arteriovenous fistula, hemodialysis access, stent, drug-eluting stent, elbow joint, chronic total occlusion

## Abstract

Cross-elbow stent placement in hemodialysis arteriovenous fistulas (AVFs) has been avoided due to risks of fracture, kinking, deformation, migration, and infection. However, elbow lesions may be refractory to angioplasty and progress to chronic total occlusion (CTO). We evaluated the safety, feasibility, and patency of cross-elbow stenting for AVF salvage. Consecutive adult hemodialysis patients who received cross-elbow stents for clinically significant AVF stenosis or CTO (September 2021 – March 2026) at a single center were retrospectively analyzed. Primary outcomes were target lesion primary patency (TLPP), access circuit primary patency (ACPP), access circuit secondary patency, and access circuit assisted primary patency, estimated by Kaplan–Meier. Technical and clinical success and complications were recorded. Forty-two patients were included; 45.2% had CTO and 57.1% had prior PTA. Eluvia-based stents were used in 83.3%. Technical and clinical success were both 100%. Mean access flow increased from 407 ± 267 to 839 ± 188 mL/min. TLPP at 3, 6, 12, and 24 months was 90.1%, 84.4%, 76.9%, and 76.9%, respectively. No stent fracture, migration, kinking, or infection occurred. Cross-elbow stenting was technically feasible and provided favorable short- to mid-term patency without device-related complications, supporting its use as a salvage option for selected refractory AVF lesions and CTOs.

## KEY MESSAGES

**What is known:** Stents crossing the elbow joint have historically been avoided because repetitive elbow motion may cause fracture, kinking, deformation, or migration; evidence for elbow-segment AVF salvage remains limited.

**What this study adds:** In 42 selected patients, cross-elbow stenting achieved 100% technical and clinical success, 76.9% TLPP at 12 months, and no observed stent fracture, migration, clinically relevant kinking, or infection.

**Potential impact:** Flexible stents, including drug-eluting stents, may be considered as a salvage option for selected refractory elbow-segment AVF stenoses and CTOs, while prospective comparative studies remain necessary.

## LAY SUMMARY

People receiving hemodialysis need a reliable arteriovenous fistula for treatment. Narrowing or blockage near the elbow can be difficult to treat, and clinicians have often avoided placing stents across the elbow because repeated bending might damage the stent. We reviewed 42 patients treated with a stent crossing the elbow. All procedures restored fistula function, blood flow improved, and about three-quarters of treated sites remained free from repeat treatment at 12 months. No stent fracture, migration, clinically important kinking, or infection was observed. These findings suggest that a flexible stent may be a useful rescue treatment for selected difficult fistula lesions, although larger prospective studies are needed.

## 1. Introduction

Durable vascular access is essential for patients receiving maintenance hemodialysis, and native arteriovenous fistulas (AVFs) remain the preferred access type because of their lower infection risk and superior long-term performance compared with prosthetic grafts and central venous catheters [1]. Nevertheless, AVF dysfunction caused by venous stenosis is common and frequently leads to inadequate dialysis, access thrombosis, repeated interventions, and eventual access abandonment.

Percutaneous transluminal angioplasty (PTA) is the first-line endovascular therapy for most clinically significant AVF stenoses, but PTA alone has important limitations in lesions characterized by elastic recoil, resistant fibrotic stenosis, venous rupture, rapid restenosis after previous angioplasty, or chronic total occlusion (CTO) [2,3]. In such circumstances, stent or stent-graft placement may be considered to provide mechanical scaffolding and maintain lumen gain [4–8]. Drug-coated balloons and drug-eluting stents have also been investigated to mitigate neointimal hyperplasia and recurrent restenosis in hemodialysis access circuits [9–11].

Lesions involving the elbow segment of a native AVF represent a distinct anatomical and biomechanical challenge. The antecubital region contains the median cubital vein, cephalic vein, basilic vein, perforator veins, and multiple venous valves and side branches. Stenosis in the venous outflow may redirect blood through deeper venous pathways *via* perforating veins and side branches [12]. Such preferential collateral or perforating flow may consequently result in poor fistula maturation, focal recurrent stenosis, or CTO.

The elbow is also exposed to repetitive flexion-extension, torsion, and compression. For this reason, stent placement across the elbow joint has historically been discouraged because of concerns regarding fracture, kinking, deformation, and migration [13–15]. Emerging evidence suggests that this historical prohibition may be overly conservative for selected patients. A recent cross-elbow stent-graft series reported acceptable 6- and 12-month patency without fracture [16]. However, available data remain sparse, particularly for elbow-segment AVF lesions and drug-eluting stents. The Eluvia fluoropolymer-coated paclitaxel-eluting stent has shown promising results in stenosed native AVFs, but its performance across the elbow joint has not been specifically reported [10,11].

Therefore, we analyzed our single-center experience of 42 hemodialysis patients who underwent cross-elbow stent placement for severe elbow-segment AVF stenosis or CTO. We aimed to assess technical feasibility, safety, short- and mid-term patency, and reintervention patterns.

## 2. Materials and methods

### 2.1. Study design and participants

This retrospective cohort study was conducted in accordance with the STROBE reporting recommendations for observational studies [17]. This study was conducted in accordance with the principles of the Declaration of Helsinki. Consecutive adult patients who underwent stent placement across the elbow joint for dysfunctional native AVFs between September 2021 and March 2026 at Sir Run Run Shaw Hospital, Zhejiang University School of Medicine, were reviewed.

Eligibility criteria for cross-elbow stent placement were: (1) a clinically dysfunctional native AVF with a severe elbow-segment stenotic or occlusive lesion without possibility of surgical revision; (2) A target lesion requiring oblique or lateral traversal across the elbow joint, as illustrated in Figure 1; (3) Single-vein stenosis or anatomically continuous lesions within the elbow joint region that were treatable within the range of one stent. (4) clinical signs of access dysfunction, including decreased dialysis pump flow, difficult postdialysis hemostasis, elevated recirculation rate, or inadequate dialysis, together with ultrasonographic confirmation of >50% diameter reduction and brachial artery access flow <500 mL/min; and (5) preservation of adequate puncture zones after stent placement. Exclusion criteria were arterial or anastomotic stenosis as the dominant lesion, unstable vital signs, severe coagulopathy, active AVF infection, clinically significant central venous stenosis, or inability to cooperate with treatment.

**Figure 1.**
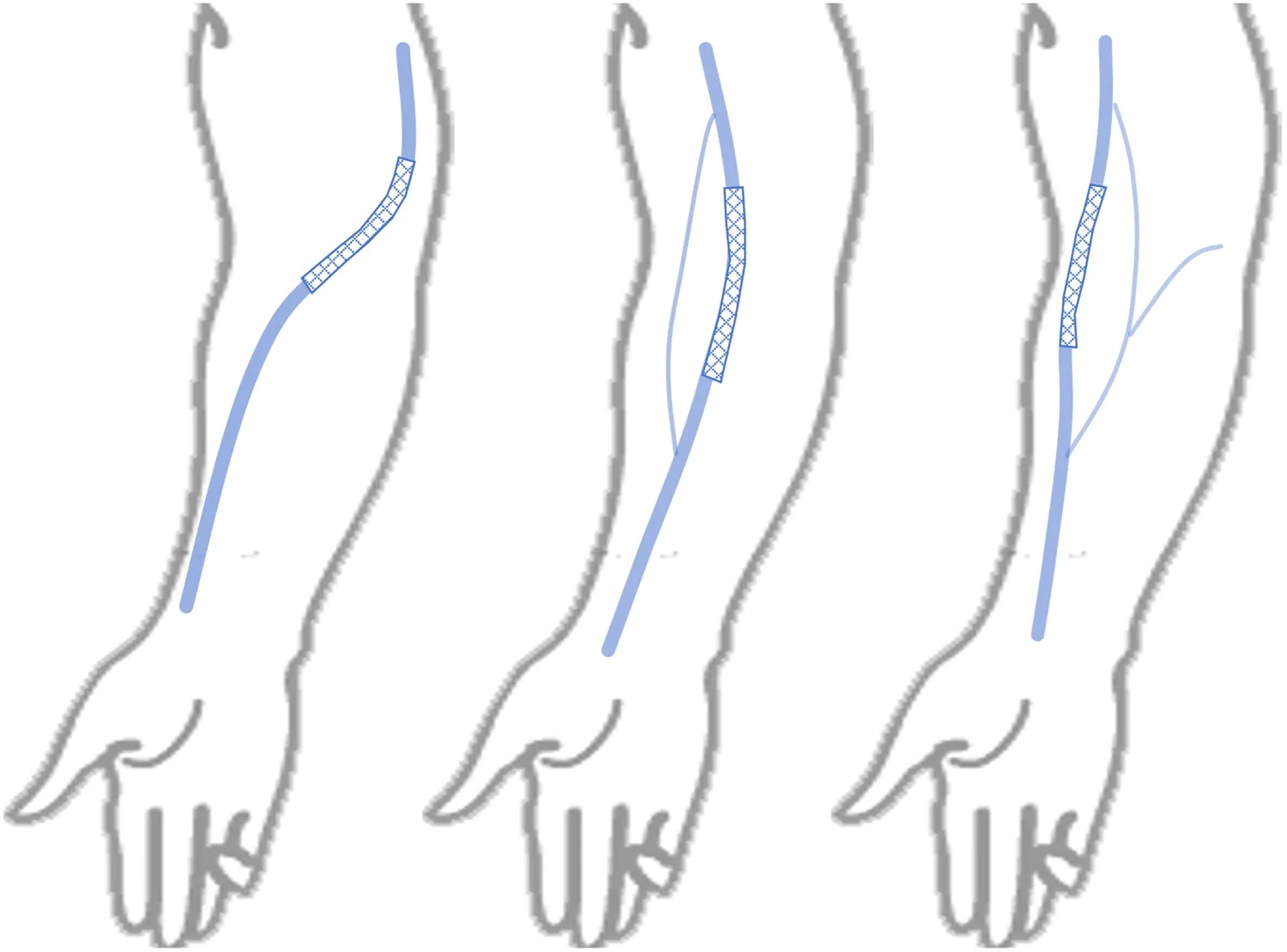
Schematic of stent placement across the elbow joint. Left: oblique crossing; middle: perpendicular crossing; right: lateral crossing. All stents traversed the elbow joint region obliquely or laterally, rather than perpendicularly.

### 2.2. Preprocedural evaluation and intervention technique

All patients were evaluated by the same vascular access team. Physical examination, duplex ultrasonography, and fistulography were used to determine lesion location, lesion length, reference vessel diameter, access flow, and the availability of future cannulation zones. All interventions were performed under both ultrasound and digital subtraction angiography guidance. CTOs were crossed using appropriate guidewires and support catheters at the operator’s discretion, with sharp recanalization performed when necessary. Predilation with high-pressure balloons was performed in most cases, followed by stent deployment to cover the entire diseased segment with margins extending into normal-caliber vein. Stent diameter was selected according to the adjacent reference vessel size, generally avoiding excessive oversizing. Postdilation was performed to optimize expansion and residual stenosis. The treated segment was planned to avoid routine cannulation zones whenever possible but could be used for buttonhole cannulation if necessary.

### 2.3. Follow-up and reintervention

Postprocedural thrill, bruit, access flow, residual stenosis, and procedural complications were recorded. Antiplatelet therapy was prescribed at the discretion of the treating physician, most commonly aspirin, clopidogrel, or dual antiplatelet therapy, according to bleeding risk, comorbidities, and stent type. All patients were followed up with regular monitoring at the dialysis unit, physical examination of the vascular access, and duplex ultrasonography performed routinely every 3 months. Additional duplex ultrasound was obtained whenever clinically indicated (e.g. decreased blood flow, elevated venous pressure, or abnormal physical findings). Reintervention was performed for recurrent clinical dysfunction with confirmatory imaging evidence of restenosis or occlusion, which was defined as the presence of clinical signs of access dysfunction, including decreased dialysis pump flow, difficult postdialysis hemostasis, elevated recirculation rate, or inadequate dialysis, together with ultrasonographic confirmation of >50% diameter reduction and brachial artery access flow <500 mL/min. To distinguish target lesion restenosis from new stenosis outside the stented segment, the following standardized definitions were used in our cohort. Target lesion restenosis was defined as >50% diameter stenosis occurring within the stent or within 5 mm of the stent edge, whereas new stenosis outside the stented segment was defined as >50% stenosis located >5 mm proximal or distal to the stent edge.

### 2.4. Definitions and outcomes

Definitions followed dialysis access reporting standards [18]. Target lesion primary patency (TLPP) was defined as the interval from stent deployment to clinically driven target lesion reintervention or access abandonment. Access circuit primary patency (ACPP) was defined as the interval from stenting to any access circuit reintervention, thrombosis, or abandonment. Access circuit secondary patency (ACSP) was defined as the interval after stenting until the access is surgically declotted, revised or abandoned. Access circuit assisted primary patency (ACAPP) was defined as the interval from stenting to the first thrombotic episode in the access circuit. Technical success was defined as successful stent implantation across the target lesion with <30% residual stenosis and restoration of access flow. Clinical success was defined as restoration of AVF function enabling hemodialysis.

### 2.5. Statistical analysis

All statistical analyses were performed using SPSS (version 26.0, IBM Corp., Armonk, NY, USA). The Shapiro–Wilk test were used to assess the normality of continuous variables. Normally distributed data were expressed as mean ± standard deviation (SD), while non-normally distributed data were presented as median with interquartile range (IQR). Categorical variables are presented as number (percentage). Pre- and postprocedural measurements were compared with paired t tests or Wilcoxon signed-rank tests, depending on distribution. Exploratory univariable logistic regression was performed for target lesion reintervention. However, due to the limited number of events, these findings should be viewed as hypothesis-generating and warrant cautious interpretation. All tests were two-sided, and *p* < 0.05 was considered statistically significant. Patency was estimated using Kaplan-Meier analysis. Kaplan-Meier survival curves were generated using GraphPad Prism (version 8.0.2; GraphPad Software, San Diego, CA, USA).

## 3. Results

### 3.1. Baseline and access characteristics

Forty-two patients met the eligibility criteria and were included in the analysis. Baseline demographic and access characteristics are summarized in Table 1. Mean age was 63.8 ± 12.5 years, and 20 patients (47.6%) were men. Twenty-four patients (57.1%) had undergone previous PTA at the target lesion, and the median number of prior PTAs was 1.0 (0.0–4.0).

**Table 1.** Baseline demographic and access characteristics.

Characteristic	Value
Patients	42
Male sex	20 (47.6%)
Age, years	63.8 ± 12.5; range 34.0–87.0
Hemodialysis vintage, years	6.0 (3.0–8.8); range 0.2–16.0
Current access age, years	5.0 (2.0–8.0); range 0.5–16.0
Etiology of ESRD	
Diabetic kidney disease	17 (40.5%)
Polycystic kidney disease	2 (4.8%)
IgA nephropathy	1 (2.4%)
Crescentic glomerulonephritis	1 (2.4%)
Amyloidosis	1 (2.4%)
Gout nephropathy	1 (2.4%)
Unknown/other	19 (45.2%)
Comorbidities	
Hypertension	37 (88.1%)
Diabetes	17 (40.5%)
Hyperuricemia	6 (14.3%)
Hyperlipidemia	12 (28.6%)
Coronary artery disease	9 (21.4%)
Peripheral vascular disease	2 (4.8%)
Atrial fibrillation	2 (4.8%)
Stroke	8 (19.0%)
Smoking	2 (4.8%)
Anticoagulant therapy	1 (2.4%)
Postprocedural antiplatelet therapy	31 (73.8%)
Aspirin + clopidogrel	13 (31.0%)
Aspirin + ticagrelor	1 (2.4%)
Clopidogrel	16 (38.1%)
Aspirin	1 (2.4%)
AVF type	
Radiocephalic fistula	38 (90.5%)
Radio-median cubital fistula	3 (7.1%)
Brachio-median cubital fistula	1 (2.4%)
Target lesion location	
Cephalic vein	8 (19.0%)
Basilic vein	4 (9.5%)
Median cubital vein	26 (61.9%)
Perforator vein	2 (4.8%)
Brachial vein	1 (2.4%)
Perforator + brachial vein	1 (2.4%)
Previous PTA	24 (57.1%)
Number of previous PTAs	1.0 (0.0–4.0); range 0.0–10.0
Interval from latest PTA, months	2.5 (1.0–4.0); range 0.2–30.0 (n = 24)

Data are presented as n (%) unless otherwise specified. ESRD, end-stage renal disease; AVF, arteriovenous fistula; PTA, percutaneous transluminal angioplasty.

### 3.2. Lesion and procedural outcomes

Lesion and procedural characteristics are summarized in Table 2. Nineteen lesions (45.2%) were CTOs, and 23 (54.8%) were severe stenoses or thrombosed stenotic lesions. Eluvia-based constructs were used in 35 patients (83.3%), whereas Covera, LifeStent, Supera, and Absolute devices were used selectively. Among the 35 patients treated with Eluvia stents, three patients (8.6%) received additional stents for non-elbow lesions; however, the elbow target lesion in each case was covered by a single Eluvia stent, and stent overlap did not involve the elbow joint region. A 7-mm stent diameter was most frequently used (34/42), and 6-cm and 8-cm stents were most commonly placed across the elbow joint (13 cases each). Predilation was performed in 41/42 cases (97.6%), with a median balloon diameter of 5.0 mm and a mean inflation pressure of 19.3 ± 5.3 atm. Postdilation was performed in all 42 cases, using a median balloon diameter of 6.0 mm and a median inflation pressure of 17.0 (14.0–20.0) atm. Median luminal diameter increased from 0.0 (0.0–1.5) mm before stenting to 5.3 (5.0–5.6) mm after stenting(*p* < 0.001, Wilcoxon signed-rank test). Mean access flow increased from 407 ± 267 mL/min before treatment to 839 ± 188 mL/min after stenting(*p* < 0.001, Paired t test). Both technical and clinical success rates were 100%, and all patients achieved adequate hemodialysis using two-needle cannulation after stent placement.

**Table 2.** Lesion and procedural characteristics.

Characteristic	Value
Target lesion characteristics	
Chronic total occlusion	19 (45.2%)
Thrombosed lesion	2 (4.8%)
Reference luminal diameter before stenting, mm	0.0 (0.0–1.5); range 0.0–3.0 (n = 34)
Percent diameter stenosis, %	100.0 (80.2–100.0); range 50.0–100.0 (n = 30)
Lesion length, cm	4.4 ± 2.7; range 0.8–12.0 (n = 23)
Preprocedural AVF flow, mL/min	407.1 ± 266.9; range 0.0–895.0 (n = 19)
Stent device	
Eluvia (including Eluvia × 2 or Eluvia + Covera)	35 (83.3%)
Covera	3 (7.1%)
LifeStent	1 (2.4%)
Supera	2 (4.8%)
Absolute	1 (2.4%)
Stent diameter, mm	
6	6 (14.3%)
7	34 (81.0%)
8	2 (4.8%)
Stent length, cm	
4	3 (7.1%)
6	13 (31.0%)
8	13 (31.0%)
10	7 (16.7%)
12	6 (14.3%)
Procedure details	
Predilation performed	41 (97.6%)
Predilation balloon diameter, mm	5.0 (5.0–5.0); range 4.0–7.0
Predilation pressure, atm	19.3 ± 5.3; range 8.0–30.0 (n = 39)
Postdilation balloon diameter, mm	6.0 (6.0–7.0); range 5.0–8.0
Postdilation pressure, atm	17.0 (14.0–20.0); range 8.0–26.0 (n = 38)
Post-stenting AVF flow, mL/min	839.3 ± 187.9; range 492.0–1167.0 (n = 19)
Luminal diameter after stenting, mm	5.3 (5.0–5.6); range 3.2–6.0 (n = 20)
Technical success	42/42 (100%)
Clinical success	42/42 (100%)
Follow-up duration, months	11.5 (5.1–26.0); range 1.0–55.0

Data are presented as n (%) unless otherwise specified. CTO, chronic total occlusion; AVF, arteriovenous fistula; PTA, percutaneous transluminal angioplasty.

### 3.3. Patency, safety, and reinterventions

Patency outcomes are shown in Figure 2. Overall TLPP at 3, 6, 12, 24, 36, and 55 months was 90.1%, 84.4%, 76.9%, 76.9%, 57.0%, and 30.8%, respectively. Overall ACPP at the same time points was 89.7%, 78.1%, 41.1%, 30.8%, 30.8%, and 30.8%. Overall ACSP was 97.4%, 97.4%, 93.2%, 93.2%, 93.2%, and 93.2%, and ACAPP was 97.4%, 97.4%, 89.6%, 84.6%, 84.6%, and 84.6%, respectively. In the Eluvia subgroup, 6- and 12-month TLPP was 93.6% and 78.2%, respectively.

**Figure 2.**
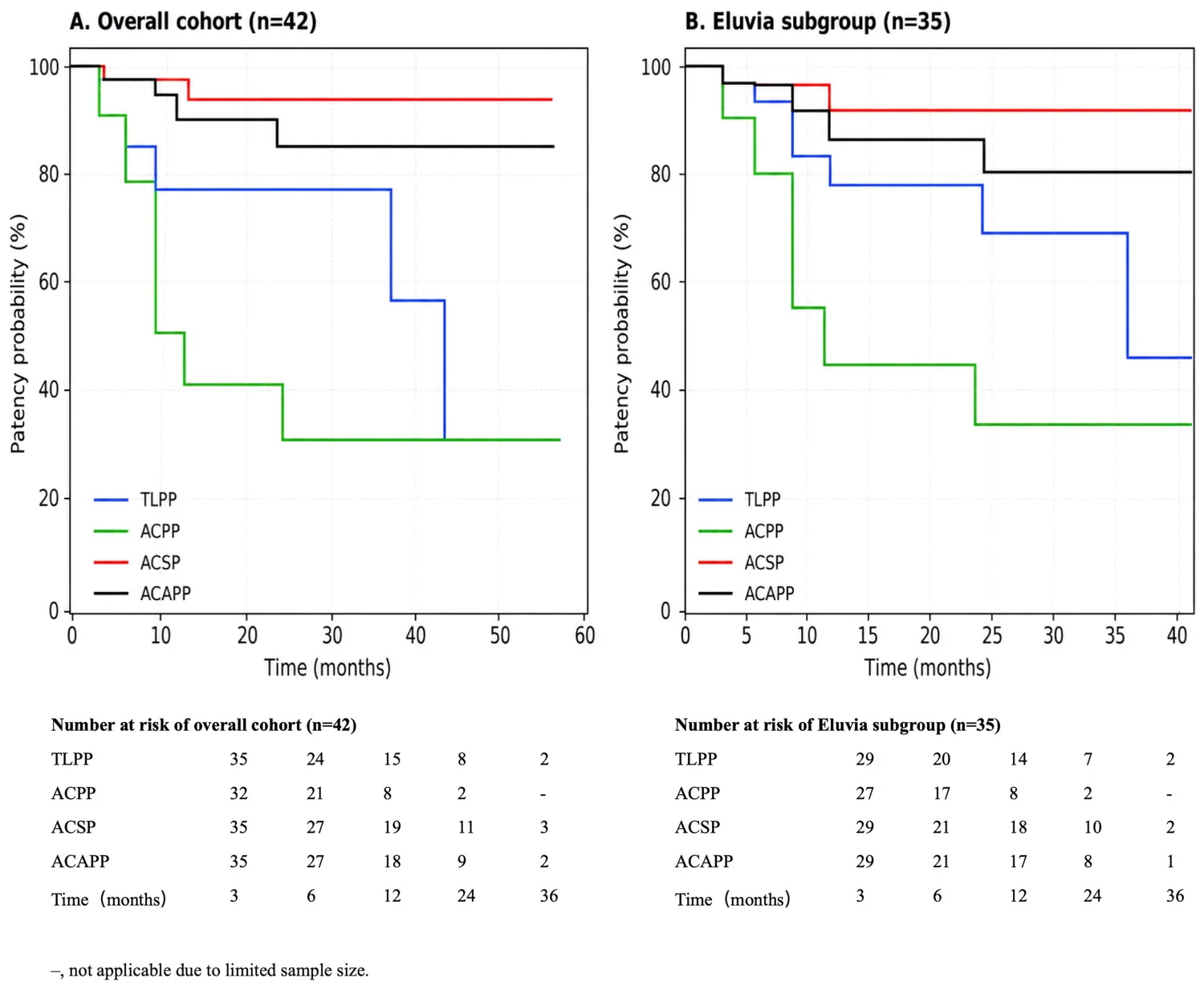
Patency outcomes after cross-elbow stent placement. A: Overall cohort (n = 42). B: Eluvia subgroup (n = 35). Curves display target lesion primary patency (TLPP), access circuit primary patency (ACPP), access circuit secondary patency (ACSP), and access circuit assisted primary patency (ACAPP).

Nine patients underwent target lesion reintervention during follow-up. Most failures were stent-edge stenoses, whereas in-stent restenosis was less frequent. No stent fracture, migration, access infection, or clinically relevant stent kinking was identified during follow-up. Dynamic sonographic assessment during elbow extension and flexion is illustrated in Figure 3, and a representative angiographic case is shown in Figure 4.

**Figure 3.**
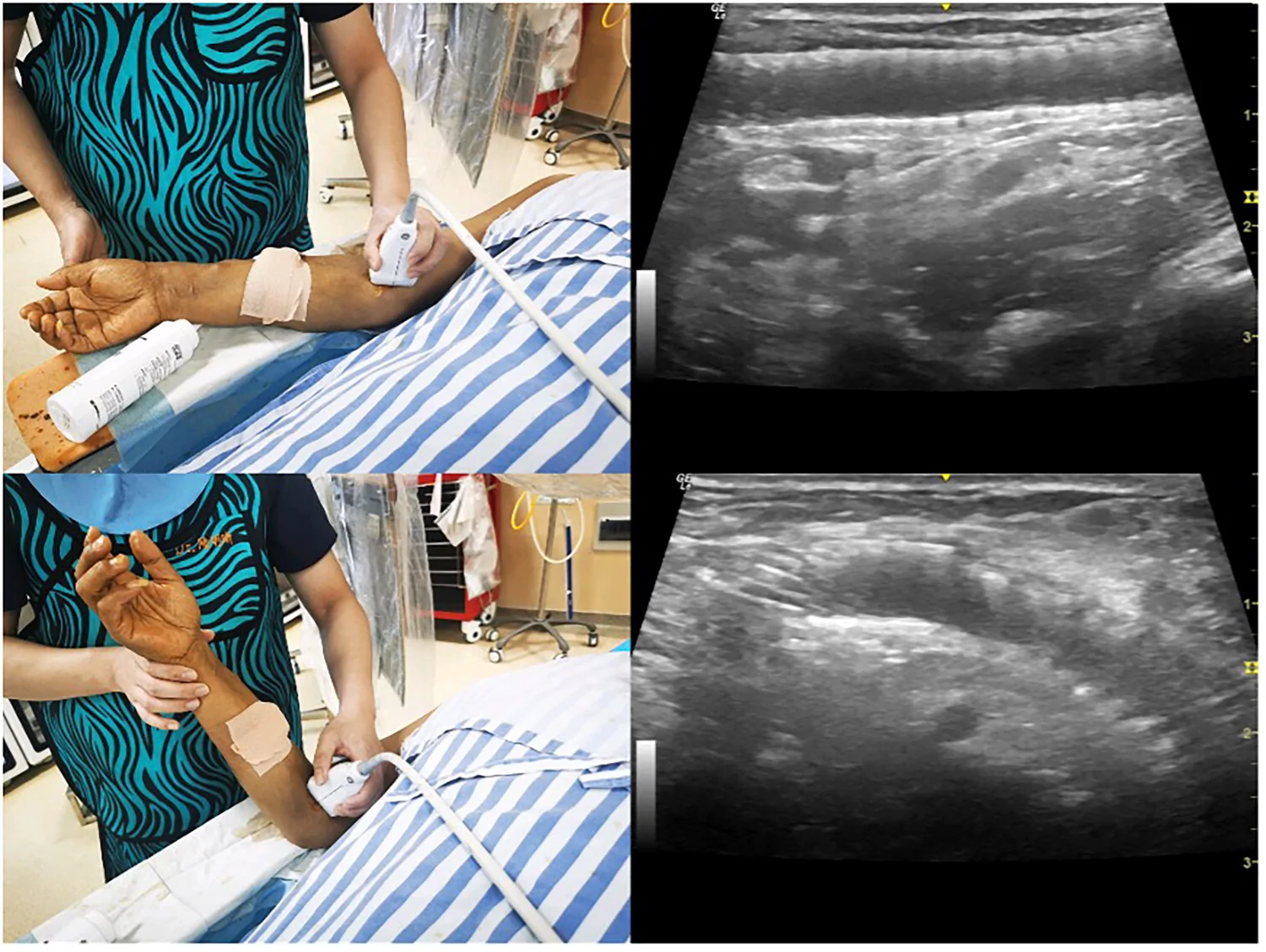
Cross-elbow stent configuration and sonographic appearance during elbow motion. Duplex ultrasound appearance of the stented segment in elbow extension and flexion, without obvious kinking or luminal collapse.

**Figure 4.**
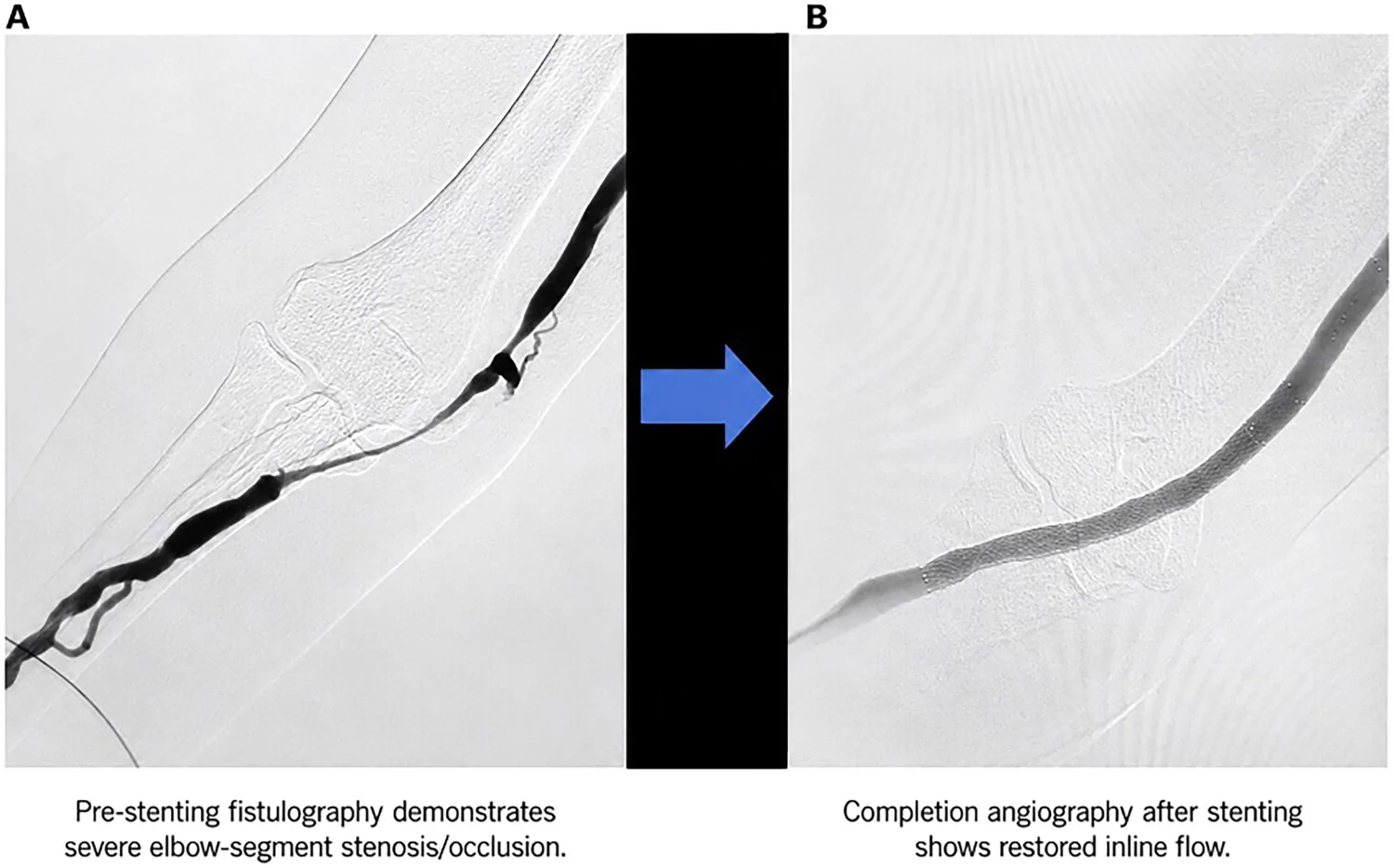
Representative angiographic case of cross-elbow stent placement. Representative fistulography before and after treatment shows severe elbow-segment stenosis/occlusion treated by cross-elbow stent deployment with restoration of brisk inline outflow and improved access function.

### 3.4. Exploratory analysis of antiplatelet therapy

Thirty-one patients (73.8%) received antiplatelet therapy after stent placement. In exploratory analysis, patients who did not receive antiplatelet therapy had a higher risk of target lesion reintervention than those who received antiplatelet therapy. This association is illustrated in Supplementary Figure 1 and detailed in Supplementary Table 1.

## 4. Discussion

This study demonstrates three clinically important findings. First, cross-elbow stent placement for severe elbow-segment AVF stenosis and CTO was technically feasible, with 100% technical and clinical success. Second, no stent fracture, migration, infection, or clinically relevant kinking was observed during follow-up, suggesting that cross-elbow stent placement can be a feasible and safe option in carefully selected hemodialysis patients with refractory AVF lesions. Third, the procedure provided favorable target lesion patency, with overall 6- and 12-month TLPP of 84.4% and 76.9%, respectively, despite a high proportion of CTOs and recurrent lesions.

The elbow segment is an underreported but clinically important site of AVF failure. In this location, venous outflow may depend on the median cubital vein, cephalic vein, basilic vein, or perforator vein. Abundant side branches and valves can create complex flow redistribution, and repeated cannulation or previous angioplasty may further promote fibrotic restenosis. In the present cohort, 45.2% of lesions were CTOs and more than half had undergone previous PTA, indicating that the treated population represented a refractory access-salvage group rather than routine *de novo* stenosis. Nevertheless, the technical and clinical success rates were both 100%, demonstrating that stent placement across the elbow joint is highly feasible in this challenging setting.

The absence of stent fracture in our cohort is particularly relevant. Several mechanisms may explain this finding. First, all stents were placed to traverse the elbow joint region obliquely or laterally, rather than perpendicularly, which may reduce focal hinge stress during elbow flexion. Second, the antecubital fossa contains soft tissue and adipose cushioning that may protect the stent from direct external compression. Third, stents were selected for favorable flexibility, fatigue resistance, and radial support. These explanations should be considered hypotheses because systematic dynamic imaging and biomechanical testing were not performed.

Our patency outcomes compare favorably with recent dialysis access stent and drug-eluting technology studies. Covered stents have demonstrated improved outcomes over PTA in selected AVF and arteriovenous graft lesions, particularly at venous anastomoses and resistant outflow stenoses [5–8]. A randomized trial showed that a drug-coated balloon can improve target lesion patency in dysfunctional AVFs, although outcomes may vary by lesion type, vessel preparation, and follow-up interval [9]. Drug-eluting stents remain less extensively studied in dialysis access, but the ELUDIA study and subsequent retrospective data suggest that paclitaxel-eluting nitinol stents may provide durable lumen support while limiting neointimal hyperplasia [10,11].

This study also provides the first dedicated clinical experience with Eluvia across the elbow joint. Eluvia was used in 83.3% of cases, and the Eluvia subgroup showed 6- and 12-month TLPP of 93.6% and 78.2%, respectively. The fluoropolymer paclitaxel-eluting design may theoretically reduce neointimal hyperplasia while preserving side-branch flow more effectively than covered stents. Moreover, an earlier study by Vogel et al. assessed SMART stent placement in 60 patients with failing grafts; among the nine patients with stents crossing the elbow joint, mean primary graft patency was 8.9 months, with only one stent fracture reported [19]. Collectively, these findings suggest that contemporary stents placed across the elbow joint can achieve acceptable patency without prohibitive fracture risk, challenging traditional avoidance of this anatomical location.

In our exploratory analysis, antiplatelet therapy appeared to be associated with a lower risk of target lesion reintervention. However, this finding should be interpreted with caution due to the small number of events and the non-randomized nature of treatment assignment. The wide confidence intervals and the potential for unmeasured confounding preclude any definitive conclusion. Therefore, this observation should be considered hypothesis-generating and warrants validation in larger, prospective studies.

This study has limitations. It was retrospective, single-center, and included only 42 patients. There was no contemporaneous PTA-only or stent-graft-only control group, and device selection was not randomized. Follow-up duration was heterogeneous, and the number of patency events was limited. Despite these limitations, the study addresses a specific anatomical question with limited published evidence and includes the largest available Eluvia-dominant cross-elbow AVF series to date.

## 5. Conclusion

Cross-elbow stent placement appears to be a feasible and safe salvage strategy for selected hemodialysis patients with refractory elbow-segment AVF stenosis or CTO. Favorable short- to mid-term TLPP and the absence of fracture or infection support reconsideration of the traditional avoidance of elbow-crossing stents. Prospective multicenter studies comparing drug-eluting stents, covered stents, drug-coated balloons, and repeat PTA are needed to define optimal device selection.

## Supplementary Material

Supplementary table 1.docx

## Data Availability

The data that support the findings of this study are available from the corresponding author upon reasonable request.
