# Supplementary material for: Cross-elbow stent placement for refractory arteriovenous fistula stenosis and chronic total occlusion in hemodialysis patients: a single-center retrospective cohort of 42 cases
Source: Ren Fail. 2026 Oct 27;48(1):2738994. doi: 10.1080/0886022X.2026.2738994 (PMC13715325; doi:10.1080/0886022X.2026.2738994)
Supplement: Supplementary file 1 [file IRNF_A_2738994_SM3377.docx]

Supplementary Table 1. Exploratory univariable logistic regression for target lesion reintervention

| **Variable** | **Odds ratio** | **95% CI** | **p value** |
| --- | --- | --- | --- |
| No postprocedural antiplatelet therapy | 5.63 | 1.15–27.44 | 0.033 |
| Previous PTA (>0 vs de novo) | 8.50 | 0.95–75.81 | 0.055 |
| Age (per year) | 0.98 | 0.93–1.04 | 0.546 |
| Male sex | 1.50 | 0.34–6.61 | 0.592 |
| Diabetes | 0.68 | 0.14–3.19 | 0.624 |
| CTO lesion | 0.53 | 0.11–2.49 | 0.422 |
| Eluvia-based construct | 0.63 | 0.10–3.92 | 0.616 |

Outcome: target lesion reintervention; event count = 9. CTO, chronic total occlusion; PTA, percutaneous transluminal angioplasty.
